# Large-scale nonlinear Granger causality for inferring directed dependence from short multivariate time-series data

**DOI:** 10.1038/s41598-021-87316-6

**Published:** 2021-04-09

**Authors:** Axel Wismüller, Adora M. Dsouza, M. Ali Vosoughi, Anas Abidin

**Affiliations:** 1 Department of Imaging Sciences, University of Rochester, Rochester, NY USA; 2 Department of Electrical and Computer Engineering, University of Rochester, Rochester, New York USA; 3 Department of Biomedical Engineering, University of Rochester, Rochester, New York USA; 4 Faculty of Medicine and Institute of Clinical Radiology, Ludwig Maximilian University, Munich, Germany

**Keywords:** Computational neuroscience, Machine learning, Computational science, Computer science, Software, Computational neuroscience

## Abstract

A key challenge to gaining insight into complex systems is inferring nonlinear causal directional relations from observational time-series data. Specifically, estimating causal relationships between interacting components in large systems with only short recordings over few temporal observations remains an important, yet unresolved problem. Here, we introduce large-scale nonlinear Granger causality (lsNGC) which facilitates conditional Granger causality between two multivariate time series conditioned on a large number of confounding time series with a small number of observations. By modeling interactions with nonlinear state-space transformations from limited observational data, lsNGC identifies casual relations with no explicit a priori assumptions on functional interdependence between component time series in a computationally efficient manner. Additionally, our method provides a mathematical formulation revealing statistical significance of inferred causal relations. We extensively study the ability of lsNGC in inferring directed relations from two-node to thirty-four node chaotic time-series systems. Our results suggest that lsNGC captures meaningful interactions from limited observational data, where it performs favorably when compared to traditionally used methods. Finally, we demonstrate the applicability of lsNGC to estimating causality in large, real-world systems by inferring directional nonlinear, causal relationships among a large number of relatively short time series acquired from functional Magnetic Resonance Imaging (fMRI) data of the human brain.

## Introduction

Identifying nonlinear and directed relations between components of a complex system, especially from simultaneously observed time series, is an actively growing area of research^1–5^. Systems with interacting components are ubiquitous in nature. A few examples of such systems are interactions between individual neurons, regions in the brain, protein interaction, climatological data, and genetic networks. However, the underlying interactions between various components of these systems are hidden, therefore, to understand their dynamics and glean more information about how various components interact or influence one another we must infer causal relations from the available observational data. For instance, analyzing signals recorded from the brain activity of healthy subjects and patients with some form of neurodegeneration can reveal vital information useful for diagnosis and treatment.

One of the most widely used approaches for estimating causal relations from time-series data is Granger causality analysis^6^. It estimates causal influence from one time series to another, if the prediction quality of the influenced time series improves when the past of an influencer time series is used, as compared to its prediction quality when the past of the influencer is not used. GC was initially formulated for linear models but later was extended to nonlinear systems in^7^ and has shown promising results. Among the alternative methods for nonlinear causal discovery, transfer entropy (TE) was introduced in^8^, which was later found to be equivalent to GC for linear Gaussian processes^9^.

In systems containing more than two time series, a bivariate analysis - i.e., considering pairs of time series solely at a time without considering the effects of confounder variables - may result in spurious causalities as a consequence of indirect connections^10^, while multivariate analysis conditioned on other variables distinguishes direct from indirect influences^11^. While GC is a multivariate analysis approach with both linear and nonlinear variants, its extension to large-scale systems, where the number of time series is much larger than the number of temporal observations, is challenging^3^, since the vector autoregressive models may involve solving inverse problems with redundant variables^12^. Various studies have proposed addressing the ill-posedness problem by dimensionality reduction or compressed sensing techniques^13,14^. Besides, most systems in nature exhibit complex dynamics that cannot be captured by linear approaches^15–17^. Nonlinear approaches may discover nonlinear relations at the cost of increased computation time and a possible increase in several parameters to be estimated.

Hence, an approach that can capture nonlinear interactions in large multivariate systems is desired. In summary, an approach that estimates interactions in multivariate systems while conditioning on all variables in the system, reducing redundancy, and being computationally feasible would be desired. Consequently, a causality analysis method should 1) be able to estimate causal interactions in multivariate systems, conditioned on all time series in the system, 2) be able to capture nonlinear dependencies, 3) work for systems with a large number of variables, and 4) be data-driven^18^. Although nonlinear extensions of GC have been proposed in^7^, and kernel-based nonlinear GC approaches in^19–21^, such approaches require a large number of observations to estimate causal relations effectively. Possible reasons for these restrictions are: besides the computational expense, the extendibility to multivariate analysis of high-dimensional dynamical systems based on a low number of temporal observations is non-trivial and involves parameter optimization of complex nonlinear time-series models on limited data. In more recent literature, methods such as multivariate transfer entropy (TE)^8^ and multivariate mutual information (MI), with nonlinear estimators such as the Kraskov-Stoegbauer-Grassberger estimator^22^, and PC-momentary conditional independence (PCMCI)^18,23^ have been developed to improve estimation of directed interactions from large-scale data. In this paper, we put forth the large-scale Nonlinear Granger Causality (lsNGC) approach to estimate the underlying directed relations between time-series. By introducing a nonlinear dimension reduction step, lsNGC aims at estimating such interactions from large complex systems, while reducing redundancies and conditioning on other variables in the system. LsNGC in addition to being a nonlinear, multivariate method, also provides control over the number of parameters to be estimated and derives significant connections in the systems. As such, lsNGC can effectively estimate interactions in large systems with short time-series data, without being computationally intensive. Besides presenting results of extensive computer simulations on synthetic networks, we also demonstrate the applicability of lsNGC on estimating connectivity from resting-state functional MRI. However, lsNGC may be useful for other domains as well, given that the data is represented as simultaneously acquired signals.

In the following sections we discuss the lsNGC algorithm and the various networks we investigate. We evaluate lsNGC against Kernel Granger causality^20^, mutual nonlinear cross-mapping methods^15^ using local models (LM), transfer entropy (TE)^8,22^, and Peter-Clark momentary conditional independence (PCMCI)^18,23^. We test the performance of simulated data with known ground truth of connections. Additionally, we demonstrate applying the proposed lsNGC approach on real time-series data recorded using functional Magnetic Resonance Imaging (fMRI) from subjects presenting with symptoms of HIV associated neurocognitive disorder (HAND) and healthy controls. If lsNGC measures can characterize brain connectivity well, it should be useful in distinguishing the two subject groups.

## Methods

### Large-scale nonlinear Granger causality

Large-scale nonlinear Granger causality adopts theoretical concepts from Granger causality analysis. Granger causality (GC) is based on the concept of time series precedence and predictability; here, the improvement in the prediction quality of a time series in the presence of the past of another time series is quantified. This reveals if the predicted time series was influenced by the time series whose past was used in the prediction, uncovering the causal relationship between the two series^6^ under investigation. The supplementary material (section 1) details the theoretical concepts involved in Granger causality analysis.

LsNGC estimates causal relationships by first creating a nonlinear transformation of the state-space representation of the time series, whose influence on others is to be measured, and another representation of the rest of the time series in the system. Consider a system with *N* time series, each with *T* temporal samples. Let the time-series ensemble ${\mathbf {X}}\in {\mathbb {R}}^{N \times T}$ be ${{\mathbf {X}}= ({\mathbf {x}}_1, {\mathbf {x}}_2, ..., {\mathbf {x}}_N)^T}$, where ${\mathbf {x}}_n \in {\mathbb {R}}^{T}$, $n \in \{1, 2, ..., N\}$, ${\mathbf {x}}_n = (x_n(1), x_n(2), ..., x_n(T))$. The time-series ensemble ${\mathbf {X}}$ can also be represented as ${\mathbf {X}}= ({\mathbf {x}}(1), {\mathbf {x}}(2), ..., {\mathbf {x}}(T))$, where ${\mathbf {x}}(t) \in {\mathbb {R}}^{N \times 1}$, $t \in \{1, 2, ..., T\}, {\mathbf {x}}(t) = (x_1(t), x_2(t), x_3(t), ..., x_N (t))^T$. Let’s say that we are interested to learn if ${\mathbf {x}}_s$ influences ${\mathbf {x}}_r$. We first construct the phase space representation of ${\mathbf {x}}_s$ with embedding dimension *d*, as $\varvec{W}_s$. The state at time *t* is $\varvec{w}_s(t) = [x_s(t-(d-1)), ~...,~ x_s(t-1),~ x_s(t)]$, and $t \in {d, ..., T-1}$. Say we are interested in quantifying the influence of ${\mathbf {x}}_s$ on ${\mathbf {x}}_r$ in the presence of all confounding variables and also by modeling nonlinearities present in the data. Confounding variables can be accounted for by performing a multivariate analysis. Additionally, we account for nonlinear interactions among time series by transforming the original space using a nonlinear transformation function.

To perform a multivariate analysis, it is desirable to have a phase space reconstruction, where prediction is performed using all the time series, apart from ${\mathbf {x}}_s$ whose influence is to be quantified. From the time-series ensemble ${\mathbf {X}}\backslash {\mathbf {x}}_s$ we construct the phase space reconstruction $\varvec{Z}_s$. The state of this multivariate system at a given time-point is

$$\begin{aligned} \varvec{z}_s(t) =\left\{ \begin{array}{ll} x_1(t-(d-1)), ..., x_1(t-1), x_1(t), ...\\ x_2(t-(d-1)), ..., x_2(t-1), x_2(t), ...\\ \quad .\\ \quad .\\ x_{N-1}(t-(d-1)), ..., x_{N-1}(t-1), x_{N-1}(t)\\ \end{array} \right. \end{aligned}$$

It should be noted that $\varvec{Z}_s$ does not contain any terms from ${\mathbf {x}}_s$.

In brief, we have constructed two systems represented by phase states $\varvec{W}_s$ and $\varvec{Z}_s$. $\varvec{W}_s$ represents the states of only the time series whose influence we want to quantify, i.e ${\mathbf {x}}_s$, and $\varvec{Z}_s$ represents the multivariate phase space incorporating all time series but ${\mathbf {x}}_s$.

Coming back to Granger causality (GC), GC works on the principle that if the prediction quality of a time series ${\mathbf {x}}_r$ improves in the presence of ${\mathbf {x}}_s$ as compared to its prediction quality in the absence of ${\mathbf {x}}_s$, having considered the rest of the time series in both models, then ${\mathbf {x}}_s$ Granger causes ${\mathbf {x}}_r$. It quantifies boost in the prediction quality, by comparing two models, one that uses information from the states of ${\mathbf {x}}_s$ and the other that does not. Let ${\mathbf {f}}$ and ${\mathbf {g}}$ represent two nonlinear functions. The two estimates of ${\mathbf {x}}_r$ are given by:

1 $$\begin{aligned} {\hat{\mathbf {x}}}_{r, s}= & {} {\mathbf {a}}_{11}{\mathbf {f}}(\varvec{Z}_s) + {\mathbf {a}}_{12}{\mathbf {g}}(\varvec{W}_s) \end{aligned}$$

2 $$\begin{aligned} {\tilde{{\mathbf {x}}}}_{r,s}= & {} {\mathbf {b}}_{1}{\mathbf {f}}(\varvec{Z}_s) \end{aligned}$$

In the above equations, ${\mathbf {a}}$ and ${\mathbf {b}}$ are the weights or model parameters, obtained by minimizing the mean squared errors in the estimate of ${\mathbf {x}}_r$. The quantities $\hat{{\mathbf {x}}}_{r, s}$ and ${\tilde{{\mathbf {x}}}}_{r,s}$ are the estimates of ${\mathbf {x}}_r$ calculated by the two models. The subscript (*r*, *s*) denotes that these models were constructed to investigate the influence of ${\mathbf {x}}_s$ on ${\mathbf {x}}_r$. In this study, we use the generalized radial basis function (GRBF) as nonlinear transformations ${\mathbf {f}}$ and ${\mathbf {g}}$. In brief, representative clusters of the state space $\varvec{Z}_s$ and $\varvec{W}_s$ are obtained using clustering methods, such as *k*-means clustering, where *k* can be seen as the number of hidden neurons in a GRBF neural network. Let $c_f$ and $c_g$ be the number of hidden layer neurons in the GRBF networks ${\mathbf {f}}$ and ${\mathbf {g}}$, respectively.

The *f*-statistic can be obtained by recognizing that the two models, equations (1) and (2), can be characterized as the unrestricted model and the restricted model, respectively. Residual sum of squares (*RSS*) of the restricted model, $RSS_{R}$, and residual sum of squares of the unrestricted model, $RSS_{U}$, are obtained. Since we are interested in testing the explanatory power of lagged values of ${\mathbf {x}}_s$ in the regression, we test the null hypothesis $H_0: {\mathbf {a}}_{12} = 0$ against the alternate hypothesis $H_a: {\mathbf {a}}_{12}$ is non-zero. This hypothesis test is performed by computing the *f*-statistic.

A measure of lsNGC can be obtained using the *f*-statistic, given by:

3 $$\begin{aligned} F_{{\mathbf {x}}_s\rightarrow {\mathbf {x}}_r} = \frac{(RSS_{R} - RSS_{U})/(p_{U} - p_{R})}{(RSS_{U})/(n - p_{U} - 1)} \end{aligned}$$

Here, $n = (T - (d-1))$ is the number of time-delayed vectors, $p_{U}$ and $p_{R}$ are the number of parameters to be estimated for the unrestricted and restricted model, respectively. For equations (1) and (2), $p_{U} = c_f + c_g$ and $p_{R} = c_f$, respectively. $F_{{\mathbf {x}}_s\rightarrow {\mathbf {x}}_r}$ quantifies the influence of ${\mathbf {x}}_s$ on ${\mathbf {x}}_r$, by testing the equality of variances of errors in prediction of the ${\mathbf {x}}_r$ by both the models i.e. equations (1) and (2). If the variance of the error in predicting ${\mathbf {x}}_r$ is lower when ${\mathbf {x}}_s$ is used, then ${\mathbf {x}}_s$ is said to Granger cause ${\mathbf {x}}_r$. The measure $F_{{\mathbf {x}}_s\rightarrow {\mathbf {x}}_r}$ is stored in the affinity matrix ${\mathbf {S}}$ at position $({\mathbf {S}})_{s,r}$, where ${\mathbf {S}}$ is an $N \times N$ matrix of lsNGC indices. Each lsNGC measure in the affinity matrix can be represented as a directed edge connecting the $s^{\mathrm {th}}$ node to the $r^{\mathrm {th}}$ node in a network graph. Implementation specifics that make lsNGC computationally efficient and various parameter information are provided in the supplementary material, section 2.

### Nonlinear transformation using generalized radial basis function

In this work we adopt the Generalized Radial Basis Functions (GRBF) neural network, originally described by^24^, with the nonlinear transformations ${\mathbf {f}}$ and ${\mathbf {g}}$. Cluster centers ${\varvec{V}^\text {T} \in {\mathbb {R}}^{c_g \times d}}$ are calculated for the state space $\varvec{W}_s$, where $c_g$ is the number of clusters obtained with *k*-means clustering. Activation function ${\mathbf {g}}$ in (5) is calculated as follows:

4 $$\begin{aligned} g_i(\varvec{w}_s(t))= \frac{e^{-{||\varvec{w}_s(t) - \varvec{v}(i)||}^2/ {\sigma }^2}}{{\sum }_{j=1}^{c_g}{e^{-{||\varvec{w}_s(t) - \varvec{v}(j)||}^2/ {\sigma }^2}}} \end{aligned}$$

where, $i \in$ {1, 2 ... $c_g$} and ${\sigma }$ is the kernel width, set to the average spacing between the centers^25^. Analogously, cluster centers ${\varvec{U}_s^\text {T} \in {\mathbb {R}}^{c_f \times (N-1)d}}$ are calculated for the state space $\varvec{Z}_s$, where $c_f$ is the number of clusters obtained with *k*-means clustering. Activation function ${\mathbf {f}}$ in (5) and (6) is calculated as follows:

5 $$\begin{aligned} f_i(\varvec{z}_s(t))= \frac{e^{-{||\varvec{z}_s(t) - \varvec{u}_s(i)||}^2/ {\sigma }^2}}{{\sum }_{j=1}^{c_f}{e^{-{||\varvec{z}_s(t) - \varvec{u}_s(j)||}^2/ {\sigma }^2}}} \end{aligned}$$

where, $i \in$ {1, 2 ... $c_f$}. The embedding dimension *d* is chosen using Cao’s method described in^26^. In this study, $c_f$ = 25 and $c_g$ = 5 is chosen empirically from preliminary analysis.

## Results

To evaluate the approach, several benchmark simulations are considered and performance is compared to four state-of-the-art approaches, mutual nonlinear cross-mapping methods^15^ using local models (LM), PC-momentary conditional independence (PCMCI)^18^, multivariate transfer entropy (TE)^8^ with the Kraskov-Stögbauer-Grassberger nonlinear estimators^22^ using the IDTxL toolbox^27^, and Kernel Granger Causality (KGC)^20^. These approaches are discussed briefly in the supplementary material, section 5. Note that various software implementations of TE are currently available in different toolboxes^28–30^. We chose IDTxL^27^, since it is the most recently developed software in this regard, providing automatic parameter selection, controlling false positives and requiring only minimal user specification.

**Figure 1:**
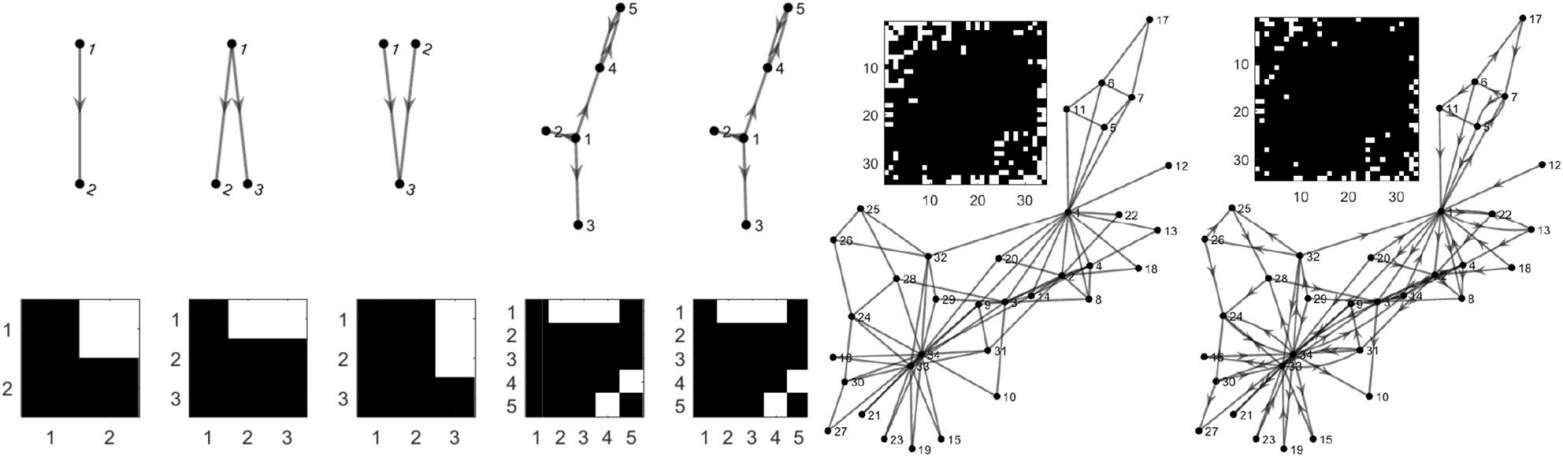
Different network structures and their corresponding adjacency matrices. Going from left to right from the first column to the seventh, we have the 2-species, 3-fan out, 3-fan in, 5-linear, 5-nonlinear, 34-Zachary1 and 34-Zachary2 networks. Generated with MATLAB R2016a^40^.

### Simulated data network models

We begin by creating benchmark simulations. Fifty different sets of each type of simulation were created, which is useful for estimating the consistency of the method. All the simulations were generated to have 500 time-points.

**Figure 2:**
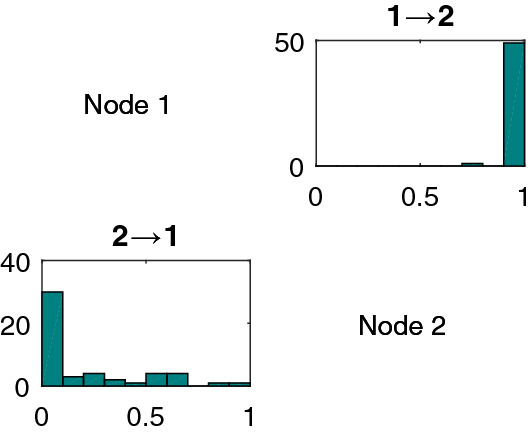
Histogram of lsNGC scores (normalized between 0 and 1) for the 2-logistic network over 50 different sets of the simulation. The influence of $x_1$ on $x_2$ is captured quite well across the 50 simulations. Generated with MATLAB R2016a^40^.

*Two species logistic model* Before investigating empirical data or systems with a large number of time series, it is imperative to test performance on a simple network structure with directed interaction. To this end, the two species logistic model which is one of the commonly studied^31^ chaotic time-series systems is considered:

6 $$\begin{aligned} \begin{aligned} x_1(t+1)=x_1(t)[r_1-r_1x_1(t)-\gamma _{1,2}x_2(t)] \\ x_2(t+1)=x_2(t)[r_2-r_2x_2(t)-\gamma _{2,1}x_1(t)] \end{aligned} \end{aligned}$$

where $r_1 = 3.7$, $r_2 = 3.8$, and $\gamma _{1,2}$ and $\gamma _{2,1}$ are the coupling constants. We adopt all values used from^15,31^. For the unidirectional case, the coupling constants take the values $\gamma _{2,1} = 0.32$, and $\gamma _{1,2} = 0$. Uniformly distributed random numbers between [0, 1] are used as initial conditions and the first 50 time points are discarded. In our results, we refer to this network as 2-logistic (Fig. 1). All the lsNGC scores are estimated using eq. 3. Figure 2 is a histogram of the lsNGC scores (normalized between 0 and 1 using min-max normalization for display purposes), assigned to $x_1 \rightarrow x_2$ and $x_2 \rightarrow x_1$ over the 50 different sets of the simulation. LsNGC is able to capture directed connections from $x_1 \rightarrow x_2$ well which is evident from Fig. 2 by the high scores assigned to $x_1 \rightarrow x_2$ compared to $x_2 \rightarrow x_1$. Detailed comparative quantitative results for the performance of various algorithms on all simulated networks shown in Fig. 1 are presented in Figs. 4, 5 and 6 .

**Figure 3:**
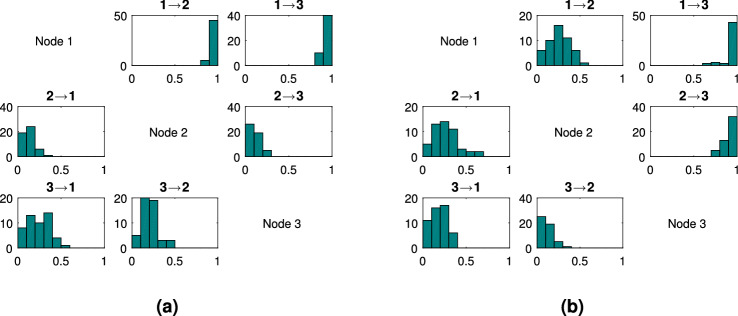
Histogram of lsNGC scores (normalized between 0 and 1) for the (**a**) 3-fan out, (**b**) 3-fan in networks over 50 different sets of the simulation. (**a**) The influence of $x_1$ on $x_2$ and $x_3$ is captured quite well across the 50 simulations. (**b**) The influence of $x_1$ and $x_2$ on $x_3$ is captured quite well across the 50 simulations. Generated with MATLAB R2016a^40^.

*Complex system with three nodes* Following this, we consider a three node complex system. See supplementary section 3 for equations. In the fan-out case, nodes $x_2$ and $x_3$ are both driven by a common source, node $x_1$, hence the dynamics of the two driven nodes contain information from $x_1$. Thus, although $x_2$ and $x_3$ do not causally influence each other, they may be correlated. Such motifs can be challenging and an approach that is able to characterize these connections well is desirable. LsNGC is able to capture the connections well and is able to recover the fan-out structure (Fig. 3a). Figure 3a, clearly demonstrates that the scores assigned across all 50 simulations by lsNGC for $x_1 \rightarrow x_2$ and $x_1 \rightarrow x_3$ are much higher than any of the other connections. Additionally, no spurious connection is estimated between $x_2$ and $x_3$. The challenge faced when estimating connections with fan-in motifs is that since $x_3$ is influenced by $x_1$ and $x_2$, detected relationships are generally weak, since the dynamics of $x_3$ is affected by two time series. From Fig. 3b, we observe that the highest strengths of connection across all 50 simulations is rightly assigned to $x_1 \rightarrow x_3$ and $x_2 \rightarrow x_3$. However, we do observe lsNGC assigns relatively high strengths ($\sim$0.5) to $x_1 \rightarrow x_2$ and $x_2 \rightarrow x_1$ for a few of the 50 simulations. We suspect this happens as a consequence of a multivariate model, since the v-structure^32^ ($x_1 \rightarrow x_3 \leftarrow x_2$) gets activated when $x_3$ is observed and information about $x_2 \ (x_1)$ is gleaned from $x_1 \ (x_2)$ if $x_3$ is observed. Nevertheless, true connection scores are higher than those the spurious connections (Fig. 3).

*Five node nonlinear network* We also generate time-series data similar to that described in^33^ using the KGC toolbox. This toolbox contains both linear and non-linear implementations of interactions between time series. Equations governing the non-linear 5-node network (5-nonlinear) and the linear system 5-node network (5-linear) are provided in the supplementary material (section 3). Results estimating direction of connection are provided in the supplementary material (section 4).

*34 node Zachary network* Systems in nature involve of a number of interacting factors. Hence, it is important to evaluate systems with a considerably large number of interacting time series. To test lsNGC on networks with a large number of nodes, we consider the Zachary dataset^34^ consisting of 34 nodes. The nodal interactions is as follows and adopted from^20^:

7 $$\begin{aligned} x_{i}(t) = \Big (1 - \sum _{j=1}^{n}{c_{i,j}}\Big )(1-ax^2_{i}(t-1)) + \sum _{j=1}^{n}{c_{i,j}}(1-ax^2_{j}(t-1)) + s\tau _{i}(t); ~i = 1,~2, ~..., ~34 \end{aligned}$$

Here, *a* = 1.8, *s* = 0.01, *c* = 0.05, where $c_{i,j}$ represents the influence *j* has on *i*, and $\tau$ is Gaussian noise with unit variance and zero mean. These quantities were adopted from^20^, where, the authors construct directed networks by assigning an edge, with equal probability of being in either direction. Apart from the directed connections, we randomly select 5 edges to be bidirectional. We construct 50 such networks and obtain 50-sets of time-series data from the corresponding network (34-Zachary2). One of the 50 networks used is shown in Fig. 1. From the generated time-series data we estimate the underlying network structure of the 50 different networks. We also construct another 50 sets of time series using the original undirected Zachary network with $c=0.025$ in equation (7) (34-Zachary1).

### Evaluating estimation of causal relations in simulations

LsNGC derives measures of nonlinear connectivity scores represented as edges in a network graph. These are non-binary scores, from which we obtain a measure of the Area Under the receiver operating characteristic Curve (AUC). However, before deriving AUC measures, the connectivity matrix is log transformed to reduce the skew in the *f*-statistic measures. The Receiver Operating Characteristic (ROC) plots the true positive rate (TPR) versus the false positive rate (FPR). Ideally, TPR = 1 and FPR = 0 at any one threshold applied on the connectivity graphs, i.e. affinity matrix, for the AUC to equal 1. An AUC of 0.5 represents assignment of random connections, analogous to guessing the absence or presence of connections. Since the AUC quantifies both, the strength of connections and the direction of information flow, it is used to evaluate performance in estimating the network structure. The AUC derives evaluation measures from the non-binarized connectivity matrix. However, it is also important to evaluate the true links obtained by significance testing of connections in the graph. The lsNGC measures of connectivity, expressed as *f*-statistic values, can be used to derive *p*-values for connections. Significant connections are obtained after multiple comparisons correction using False Discovery Rate (FDR) method at $p < 0.05$. From the thresholded affinity matrix, measures of *sensitivity* and *specificity* are derived.

**Figure 4:**
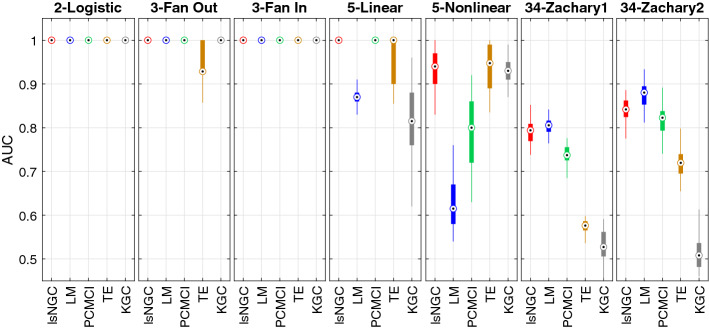
AUC results for the various networks comparing different methods, visualized as boxplots. Figure generated with MATLAB R2016a^40^. The bottom end of the box represents the first quartile and the top of the box represents the third quartile. The circle with a dot in the box represents the distribution median. A general trend that is noticeable here is that the performance drops for all approaches as the number of nodes increases. Note that lsNGC performs competitively for all networks.

**Figure 5:**
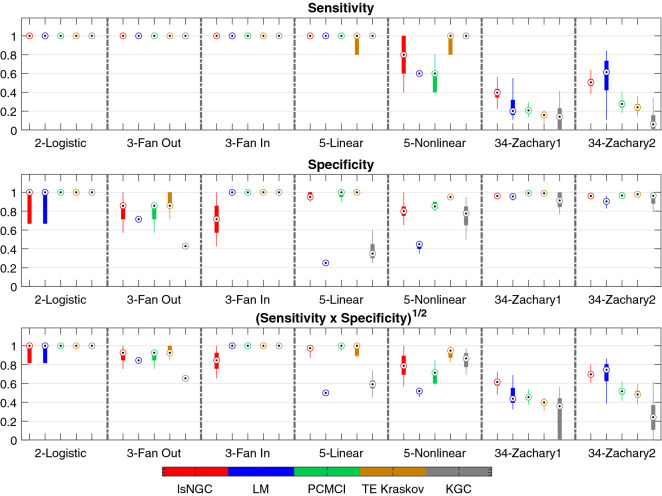
Sensitivity and specificity results for the three approaches across all networks, visualized as boxplots. Figure generated with MATLAB R2016a^40^. The bottom end of the box represents the first quartile and the top of the box represents the third quartile. The circle with a dot in the box represents the distribution median. We also plot a measure combining the two measure to estimate overall performance. In general, we observe lsNGC works consistently well for all networks.

Here, we present quantitative results on the recovered graph for the various simulations. For every network investigated in this study, 50 different sets of time series were simulated. Results are summarized as boxplots (example: Figs. 4, 5). The circle with a dot inside the box represents the distribution median. The box spans the first quartile to the third quartile which is its interquartile range (IQR). The vertical extensions from the box, whiskers, have a maximum length of 1.5 times the IQR. The median of the AUC, sensitivity and specificity are represented as ${\tilde{AUC}}_{method}$, ${\tilde{sens}}_{method}$ and ${\tilde{spec}}_{method}$, respectively, where *method* refers to the analysis method, i.e., lsNGC, LM, PCMCI, TE or KGC.

Results in Fig. 4 were obtained for all network structures in Fig. 1 generated with 500 time-points. In this figure, red, blue, green, orange and grey correspond to lsNGC, LM, PCMCI, TE and KGC respectively. All the approaches work very well for the smaller networks i.e. 2-logistic, 3-Fan out and 3-Fan-in networks, other than ${\tilde{AUC}}_{TE} = 0.93$ for the 3-fan out network. However, KGC’s and LM’s performance drops when using the linear system with 5 nodes, with a ${\tilde{AUC}}_{KGC} = 0.82$ and ${\tilde{AUC}}_{LM} = 0.87$, compared to ${\tilde{AUC}}_{lsNGC} = 1$, ${\tilde{AUC}}_{PCMCI} = 1$ and ${\tilde{AUC}}_{TE} = 1$. Additionally, LM performs poorly for the nonlinear 5 node network ${\tilde{AUC}}_{LM} = 0.62$, followed by PCMCI, ${\tilde{AUC}}_{LM} = 0.8$, while lsNGC, TE and KGC perform comparably. For both, directed and undirected 34-node Zachary networks, performance of all methods drop compared to their performance for smaller networks. However, lsNGC, LM and PCMCI show superior performance compared against TE and KGC with KGC showing poorest performance. KGC performs poorly, with most recovered networks being random at medians of 0.52 and 0.51 for the networks. These results demonstrate that KGC cannot capture right connections for a relatively large network with just few time-points. In their original paper^20^, the authors tested the Zachary network, but with 10,000 time-points; which is an unrealistic scenario for most practical applications. Refer to Table 1 in supplementary material for detailed AUC values.

Given, the *f*-statistic for the lsNGC measure, we obtain significant connections amongst the lsNGC derived estimates as described in “Large-scale nonlinear Granger causality” section. Figure 5 plots the sensitivity, specificity and a combination of the two. Here, we observe that for small networks with 2-3 nodes, all approaches perform well with the exception of KGC for the fan-out network. TE shows the best performance for small networks. For the 5-node networks, LM performs quite poorly, whereas overall, TE does well closely followed by lsNGC. Large networks, such as the Zachary network with 34 nodes, are generally difficult to recover since the total number of possible connections grows as a function of $N(N - 1)$. Here, we observe that KGC is the poorest of the methods tested, followed by TE and PCMCI. LM and lsNGC are comparable. From Figs. 4 and 5 , we observe that lsNGC performs consistently well for the various networks topologies. Additionally, it should be stressed that significant connections should be estimated to obtain a thresholded network graph. As we demonstrated in equation 3, this is straightforward to calculate with the lsNGC formulation. However, obtaining significant connections for LM, and TE, is computationally expensive since it requires the calculation of surrogate time series, followed by obtaining a null distribution using the various methods. For more details refer to the supplementary material section 6. Next we evaluate the effect of time-series length on network graph estimation.

**Figure 6:**
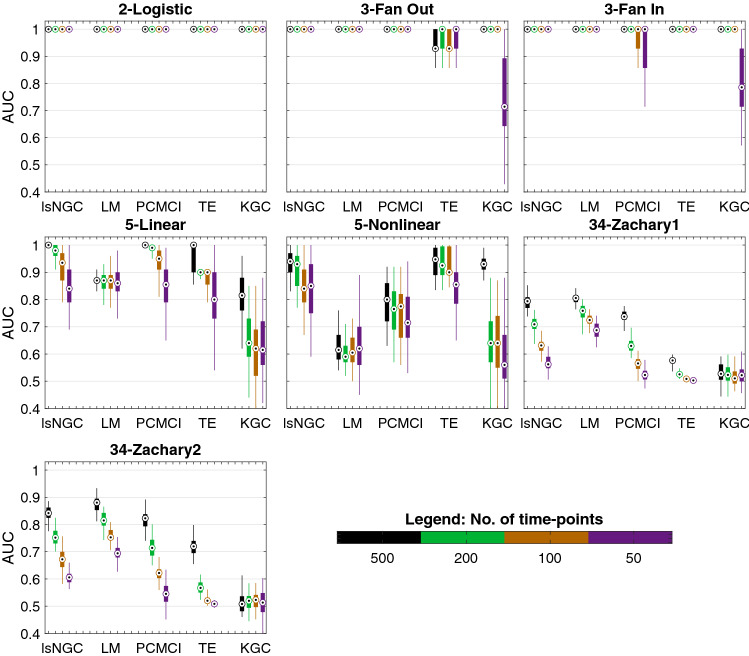
Effect of time-series length on performance. We observe that for large networks, KGC and TE do not perform well. LsNGC, on the other hand, performs well for all networks. The results are visualized as boxplots. The bottom end of the box represents the first quartile and the top of the box represents the third quartile. The circle with a dot in the box represents the distribution median. Generated with MATLAB R2016a^40^.

Due to various constraints, such as cost, sensor limitations, manpower, time, etc., it is not always feasible to collect a large number of observations (time-points) for the factors under investigation. Thus, it is also essential to test the performance of lsNGC for a lower number of observations. To this end, the time-series length is varied from 500 to 50 time-points. Figure 6 compares AUC results across methods for decreasing number of time-points. Comparing performance across methods for all the networks, we see that lsNGC consistently performs well. For small networks, having 2-3 nodes, all methods perform comparably; however, a sudden drop in performance of KGC occurs when the time-series length reduces to 50 time-points for the 3 node networks. TE performs the worst in for the fan-out motif. We also observe a gradual drop in PCMCI performance with decreasing time-series length for the fan-in motif. For the 5-node networks, it is interesting to note that the sudden drop in KGC’s performance is observed much earlier, at 200 time-points, for the 5-node network, while for the 34-node Zachary network, its performance oscillates across a median AUC of 0.5, indicating detection of random connections for 500 to 50 time-points. LsNGC, PCMCI, and TE have comparable performance for the 5-linear network. However, PCMCI shows a drop in performance for the 5-nonlinear network. TE struggles with reaching good performance for the 34 node network, while PCMCI, although quite comparable to lsNGC, shows a steeper drop in performance with a reduction in time-series length. LsNGC and LM perform equally well for the networks with 2-3 nodes across different time-series lengths. For the 5-node network, results indicate that LM reaches a bottleneck in performance, and is not able to improve as much as lsNGC, PCMCI and TE with increased time-series length. Nevertheless, it is important to note that graph structure recovered with LM does not degrade with decreasing time-series lengths. LM does not perform well for the 5-node nonlinear network. Additionally, we investigate both undirected (34-Zachary1) and directed networks (34-Zachary2). The drop in performance of lsNGC is markedly steeper than that of LM with decreasing time-series lengths. However, its performance is comparable to LM at $T > 200$. Additional experiments (results not included) when multivariate mutual information with the Kraskov algorithm^22^, showed that the method performed poorly when compared to TE.

### Functional magnetic resonance imaging data

LsNGC showed promising results on the simulations. Nevertheless, its performance on real data can give more insight into its usability for various applications involving estimation of underlying interactions from signals. In this work, we analyze its performance on functional Magnetic Resonance Imaging (fMRI) data. It has been demonstrated that individuals presenting with symptoms of HAND have quantifiable differences in connectivity^35,36^ from controls. We hypothesize that if lsNGC can capture brain connectivity from fMRI data for the subjects well, differences in connectivity between subjects with HAND and controls should be observed. Hence, we tested how well a classifier was able to discriminate the two subject groups. The classifier was able to learn relevant differences from the two groups using connectivity derived with lsNGC (AUC = 0.88 and accuracy = 0.77), suggesting that lsNGC was able to characterize the interactions well. More details on the data and analysis approach can be found in supplementary material section 7.

## Discussion

The paper puts forth a novel approach called large-scale nonlinear Granger causality (lsNGC). The lsNGC unveils the underlying nonlinear interactions among multivariate and large-scale time series. We demonstrate its applicability on the real and synthetic data and its advantages for systems with large nodal and short temporal measurements. A common trend across all investigated methods, i.e. lsNGC, LM, PCMCI, TE and KGC is the decline in performance with an increasing number of nodes, for a given number of temporal observations. However, performance may be improved by increasing the number of observations. We observe that lsNGC outperforms other approaches in most cases. Furthermore, KGC and TE are markedly more susceptible to poor performance with increased number of nodes (time series, Fig. 6) compared to lsNGC. When increasing the number of nodes in the network, the number of time-points has to be significantly increased to produce meaningful results with KGC. Although, lsNGC’s performance gradually drops with decreasing number of time-points, for all practical lengths of the time-series data, it outperforms KGC and TE, making lsNGC more reliable for larger systems with fewer time-points. Comparing lsNGC with LM, it is seen that the directed interactions recovered with LM does not degrade as rapidly with decreasing time-series lengths. This can be attributed to the lower complexity of LM, hence fewer parameters to be estimated compared to lsNGC. As such, given very short time series, LM may be able to outperform lsNGC. However, the low complexity of LM results in models with high bias. Such high bias comes at the price of its significant performance drop as seen in the 5-node nonlinear network. To put it simply, LM is a low variance, high bias model, whereas lsNGC is a higher variance, lower bias model. This becomes more evident when analyzing the network with 34 nodes. The drop in performance of lsNGC is markedly steeper than that of LM with decreasing time-series lengths. However, its performance is comparable to LM at $T > 200$. Compared with PCMCI, lsNGC performs better for systems with a large number of nodes and is not as strongly affected by reduction in the number of time points as PCMCI. A possible reason could be that, although PCMCI has been proposed to analyze large-scale multivariate time series, during the two-stage process of the algorithm, if any of the PC-stage or MCI-stage fault, the results may be changed, which may be more evident for short time series with a large number of variables. Additionally, in our simulations, we use the partial correlation for the PC-stage’s independence tests, where it is noteworthy that partial correlation is still vulnerable to confounding variables of third and beyond momentum. TE also performs very poorly in large systems as seen from the results on 34-node Zachary networks. TE with the Kraskov estimator approximates the probability distribution based on the k-nearest neighbors^27^. The Kraskov method is a non-parametric approach for density estimation, which approximates the probability distribution based on the k-nearest neighbors and has been used to obtain TE in^27^. However, for enhancing accuracy in large-scale multivariate time series, one needs to increase the number of nearest neighbors, which enforces a tradeoff between computational cost and accuracy for density estimation. Newer parametric estimators, such as the Mutual Information Neural Estimator (MINE)^37^, that rely on the characterizing the mutual information as the Kullback-Leibler divergence, may improve the performance of TE and possibly lsNGC (here we use GRBF as a density estimator). However, such an estimator requires training a generative model without any explicit assumptions about the underlying data distribution. While this estimator is promising, we anticipate it to be quite computationally intensive. With lsNGC, we focus on an approach that can estimate density using a closed form solution in the OLS formulation, unlike MINE, which requires learning of a representation using an iterative/gradient descent learning.

The success of lsNGC on simulated data motivated us to test its performance on real data. To this end, we evaluated the connectivity matrices derived using lsNGC on real fMRI data from healthy controls and subjects with HIV associated neurocognitive disorder (HAND). The connectivity measures used as features in a classifier were highly discriminative. This suggests that lsNGC is able to capture relevant information regarding interaction between different regions in the human brain.

LsNGC is formulated as a multiple regression problem with nonlinear basis transformation using GRBF with $(T - (d-1))$ samples in the regression task. Evidently, the larger the number of samples, higher the power of the hypothesis tests. An advantage of lsNGC’s formulation is that it allows for control over the number of predictors regardless of the number of nodes in a network, by adjusting the number of cluster centers, $c_f$ and $c_g$ parameters, which determine the number of predictors for the regression task. Moreover, the formulation of lsNGC can be directly used to estimate significant connections, using the *f*-statistic, without creating a null distribution from surrogate time series as is commonly done. Furthermore, using the *f*-statistic, our results on sensitivity and specificity demonstrate that lsNGC performs very well compared to other methods. Obtaining relevant connections after creating a null distribution with surrogate time series is possible with lsNGC; however, it will significantly increase the computational cost. The flexibility of estimating significant connections with lsNGC is a significant advantage over traditional approaches for detecting causality. Nevertheless, it is understandable to err on the side of caution, since estimating measures of significance with the *f*-statistic requires conditions of independence between delay embeddings to be met. Granger causality assumes that time series influence each other only *d* points in the past. Poor estimation of the order ’*d*’ can result in erroneous values of significance. This is especially relevant when time series in the system are themselves dependent (temporal autocorrelation), as is commonly the case with fMRI data. Recent work propose hypothesis tests under autocorrelation^38,39^. Experimental evidence in^39^ demonstrates that commonly used hypothesis tests may result in type I or type II errors if autocorrelations exist among the various components in a system. This an important problem to consider and it is worth investigating the effect of autocorrelated time series on the lsNGC derived f-statistic measure, in a subsequent study.

Although, lsNGC shows promising performance for real world data and simulations, one of its shortcomings is that its formulation only allows for additive relationship between the time series whose influence is to be estimated and every other time series. In brief, lsNGC is additive in the functions ${\mathbf {f}}$ and ${\mathbf {g}}$. An additional term accounting multiplicative relationships will address this drawback, while potentially increasing the complexity of the model. In the future, we plan to investigate the effect of such a term on inferring directed dependence. In this study, we selected a fixed number of hidden neurons for all the analysis. However, these parameters can be modified without significant changes to performance, as long as they are large enough to represent the system well and small enough such that the complexity of the predictor is not increased compared to the number of time-points.

In summary, lsNGC is robust in inferring causal, nonlinear interactions across different network topologies. Like all investigated methods, underlying network size does affect its performance; however, it significantly outperforms conventionally used methods for practical time-series lengths (Fig. 6). LsNGC benefits from being a nonlinear, multivariate method, whose formulation provides control over the number of estimated parameters and derives significant connections in networks. As such, lsNGC can effectively infer measures of directed dependence from large systems of short multivariate time series without being computationally intensive.

## Conclusion

In this work, we propose a nonlinear method for large-scale multivariate time series, named large-scale nonlinear Granger causality (lsNGC), to infer underlying directed interactions from time-series data. The number of temporal observations limit most approaches proposed in the literature and imposes a challenge for performing multivariate, nonlinear causality analysis to reveal the underlying interactions of large systems. We investigated some of the existing nonlinear causal inference methods’ advantages and limitations through experimentation and analysis with different network structures. We demonstrated the advantage of lsNGC over current state-of-the-art multivariate and bivariate approaches. The high AUC, good sensitivity and specificity results for various lengths of time-series data demonstrate its potential and applicability to real world data. Furthermore, lsNGC’s formulation allows obtaining binary interactions without creating a null distribution from surrogate time series, which is computationally expensive, especially for large networks. Finally, we have demonstrated the applicability of lsNGC in inferring interactions among different regions of the brain from brain activity data obtained using functional magnetic resonance imaging (fMRI). Besides clinical applications for diagnosing neurological disorders, such an approach may reveal valuable insights about directed interactions in the brain.

## Supplementary Information

Supplementary Information.
