# Supplementary material for: Large-scale nonlinear Granger causality for inferring directed dependence from short multivariate time-series data
Source: Sci Rep. 2021 Apr 9;11:7817. doi: 10.1038/s41598-021-87316-6 (PMC8035412; doi:10.1038/s41598-021-87316-6)
Supplement: Supplementary file 1 — Supplementary Information. [file 41598_2021_87316_MOESM1_ESM.pdf]

Supplementary material for - Large-scale nonlinear Granger causality for inferring directed dependence from short multivariate time-series data

Axel Wismüller[1,2,3,4,a], Adora M. DSouza [2,a,*], M. Ali Vosoughi[2*], Anas Z. Abidin[3]

[1]Department of Imaging Sciences, University of Rochester, NY, USA, [2]Department of Electrical and Computer Engineering, University of Rochester, Rochester, New York, USA, [3]Department of Biomedical Engineering, University of Rochester, Rochester, New York, USA, [4]Faculty of Medicine & Institute of Clinical Radiology, Ludwig Maximilian Univ, Munich, Germany
[a]These authors contributed equally to this work
[*]Corresponding author (adora.dsouza@rochester.edu)

1 Granger causality analysis

The principle of Granger causality (GC) is based on the concept of precedence and predictability, where the improvement in prediction quality of a time-series in the presence of the past of another time-series is evaluated and quantified, revealing the directed influence between the two series[1] investigated. As lsNGC is an extension of traditional multivariate GC (mvGC), the basic concepts of mvGC are briefly described here.

Consider a system with N time-series, each with T temporal samples. Let the time-series ensemble $\mathbf{X} \in \mathbb{R}^{N\times T}$ be $\mathbf{X} = (\mathbf{x}_1, \mathbf{x}_2, ..., \mathbf{x}_N)^T$, where $\mathbf{x}_n \in \mathbb{R}^T$, $n \in \{1,2,...,N\}$, $\mathbf{x}_n = (x_n(1), x_n(2), ..., x_n(T))$. The time-series ensemble $\mathbf{X}$ can also be represented as $\mathbf{X} = (\mathbf{x}(1), \mathbf{x}(2), ..., \mathbf{x}(T))$, where $\mathbf{x}(t) \in \mathbb{R}^{N\times 1}$, $t \in \{1,2,...,T\}, \mathbf{x}(t) = (x_1(t), x_2(t), x_3(t), ..., x_N(t))^T$.

We use multivariate vector auto-regression which is the most common prediction scheme used in GC analysis[2,3].

$$\mathbf{x}(t) = \sum_{j=1}^{d} \mathbf{A}_j \mathbf{x}(t-j) + \mathbf{e}(t) \tag{1}$$

Here, the matrices $\mathbf{A}_j$ are the model parameters obtained by minimizing the mean squared errors in the estimate of $\mathbf{X}$, where $\mathbf{A}_j$ is an $N\times N$ matrix with $j \in 1,2,...,d$, and d is the lag order. We define $\hat{\mathbf{X}}$ as the predicted system (1) without the error term, and $\mathbf{E}$ as the set of residuals defining the difference between the actual and predicted values of $\mathbf{X}$.

$$\hat{\mathbf{x}}(t) = \sum_{j=1}^{d} \mathbf{A}_j \mathbf{x}(t-j) \tag{2}$$

$$\mathbf{E} = \mathbf{X} - \hat{\mathbf{X}} \tag{3}$$

Granger causality (GC) analysis establishes a causal influence score from time-series $\mathbf{x}_s$ to $\mathbf{x}_r$ on the premise that, if the predictability of time-series $\mathbf{x}_r$ improves in the presence of the past of time-series $\mathbf{x}_s$, then $\mathbf{x}_s$ Granger causes $\mathbf{x}_r$. GC analysis estimates causal relationships in a multivariate sense by considering the full ensemble of time-series including confounding time-series that neither represent $\mathbf{x}_s$ nor $\mathbf{x}_r$. We obtain the influence of $\mathbf{x}_s$ on $\mathbf{x}_r$ by quantifying the reduction in prediction quality of $\mathbf{x}_r$ in the absence of time-series $\mathbf{x}_s$. Equations (2) and (3) obtain the prediction of all series when the full ensemble of time-series is used. Next, we obtain the prediction error $\mathbf{E}_{\mathbf{X}\setminus\mathbf{x}_s}$, when series $\mathbf{x}_s$ is removed from the ensemble $\mathbf{X}$.

If the prediction quality of $\mathbf{x}_r$ is higher when $\mathbf{x}_s$ is used (2) rather that when it is not used for predicting time-series $\mathbf{x}_r$, then it is said that $\mathbf{x}_s$ Granger causes $\mathbf{x}_r$. The f-statistic can be obtained by recognizing that the two models can be characterized as the unrestricted model and the restricted model, where the unrestricted model is eq. (2), and the restricted model is constructed in the absence of time-series $\mathbf{x}_s$. Residual sum of squares (RSS) of the restricted model, RSS_R, and residual sum of squares of the unrestricted model RSS_U are obtained. n is the number of observations in the regression, q_U and q_R are the number of parameters to be estimated for the unrestricted and restricted model, respectively. For traditional multivariate GC, $q_R = d\times(N-1)$ and $q_U = d\times N$, respectively.

A measure of GC can be obtained using the f-statistic, given by:

$$FGC_{\mathbf{x}_s\to\mathbf{x}_r} = \frac{(RSS_R - RSS_U)/(q_U - q_R)}{(RSS_U)/(n - q_U - 1)} \tag{4}$$

$FGC_{\mathbf{x}_s\to\mathbf{x}_r}$ quantifies the influence of $\mathbf{x}_s$ on $\mathbf{x}_r$, by testing the equality of variances of errors in prediction of the $\mathbf{x}_r$ by both the models. If the variance of the error in predicting $\mathbf{x}_r$ is lower when $\mathbf{x}_s$ is used, then $\mathbf{x}_s$ Granger causes $\mathbf{x}_r$. Significant interactions can be obtained using the f-statistic.

2 Implementation specifics for large-scale Nonlinear Granger causality (lsNGC)

2.1 Implementation specifications

Nonlinear Granger causality analysis has been investigated and theoretical work laying the foundation of mathematical formulation to perform such an analysis has been studied[4–6]. However, while sound theoretical concepts are prerequisite, practical implementation, especially for large systems having many nodes (time-series) is not always straightforward. In this section, we briefly describe implementation specifications that increase scalability and reduce computation time significantly.

Let us say that we are interested to learn if $\mathbf{x}_s$ influences $\mathbf{x}_r$. We first construct the phase space representation of $\mathbf{x}_s$ with embedding dimension d, as $\boldsymbol{W}_s$. The state at time t is $\boldsymbol{w}_s(t) = [x_s(t-(d-1)),\ldots,x_s(t-1),x_s(t)]$, and $t \in d,\ldots,T-1$.

To perform a multivariate analysis, a phase space reconstruction is constructed, where prediction is performed using all the time-series apart from $\mathbf{x}_s$ whose influence is to be quantified. From the time-series ensemble $\mathbf{X}\backslash\mathbf{x}_s$ we construct the phase space reconstruction $\boldsymbol{Z}_s$. The state of this multivariate system at a given time-point is

$$\boldsymbol{z}_s(t) = \begin{cases} x_1(t-(d-1)),\ldots,x_1(t-1),x_1(t),\ldots \\ x_2(t-(d-1)),\ldots,x_2(t-1),x_2(t),\ldots \\ \quad\quad . \\ \quad\quad . \\ x_{N-1}(t-(d-1)),\ldots,x_{N-1}(t-1),x_{N-1}(t) \end{cases}$$

It should be noted that $\boldsymbol{Z}_s$ does not contain any terms from $\mathbf{x}_s$. Let $\mathbf{f}$ and $\mathbf{g}$ represent two nonlinear functions. The two estimates of $\mathbf{X}$ are given by:

$$\hat{\mathbf{X}} = \mathbf{A}_1\mathbf{f}(\boldsymbol{Z}_s) + \mathbf{A}_2\mathbf{g}(\boldsymbol{W}_s) \tag{5}$$

$$\tilde{\mathbf{X}}\backslash\mathbf{x}_s = \mathbf{B}_1\mathbf{f}(\boldsymbol{Z}_s) \tag{6}$$

In the above equations, $\mathbf{A}_1$, $\mathbf{A}_2$ and $\mathbf{B}_1$ are the weights or model parameters, obtained by minimizing the mean squared errors in the estimate of all time-series in $\mathbf{X}$. The time-series $\hat{\mathbf{x}}_{r,s}$ from $\hat{\mathbf{X}}$ and $\tilde{\mathbf{x}}_{r,s}$ from $\tilde{\mathbf{X}}\backslash\mathbf{x}_s$ are the estimates obtained for $\mathbf{x}_r$. The subscript (r,s) denotes that these are estimates of $\mathbf{x}_r$ obtained to investigate the influence of $\mathbf{x}_s$ on $\mathbf{x}_r$. The two constructed models, given in eq. (5) and (6), are the unrestricted and restricted model, respectively. The RSS of the two models gives us an f-statistic measure. In this study, we use the generalized radial basis function (GRBF) neural network as nonlinear transformations $\mathbf{f}$ and $\mathbf{g}$.

2.2 Nonlinear transformation using Generalized Radial Basis Function

In this work we adopt the Generalized Radial Basis Function (GRBF), originally described by[7], as the nonlinear transformation $\mathbf{f}$ and $\mathbf{g}$. Cluster centers $\boldsymbol{V}^{\mathrm{T}} \in \mathbb{R}^{c_g \times d}$ are calculated for the state space $\boldsymbol{W}_s$, where c_g is the number of clusters obtained with k-means clustering. Activation function $\mathbf{g}$ in (5) is calculated as follows:

$$g_i(\boldsymbol{w}_s(t)) = \frac{e^{-||\boldsymbol{w}_s(t)-\boldsymbol{v}(i)||^2/\sigma^2}}{\sum_{j=1}^{c_g} e^{-||\boldsymbol{w}_s(t)-\boldsymbol{v}(j)||^2/\sigma^2}} \tag{7}$$

where, $i \in \{1,\ 2\ \ldots\ c_g\}$ and σ is the kernel width, set to the average spacing between the centers[8]. Analogously, cluster centers $\boldsymbol{U}_s^{\mathrm{T}} \in \mathbb{R}^{c_f \times (N-1)d}$ are calculated for the state space $\boldsymbol{Z}_s$, where c_f is the number of clusters obtained with k-means clustering. Activation function $\mathbf{f}$ in (5) and (6) is calculated as follows:

$$f_i(\boldsymbol{z}_s(t)) = \frac{e^{-||\boldsymbol{z}_s(t)-\boldsymbol{u}_s(i)||^2/\sigma^2}}{\sum_{j=1}^{c_f} e^{-||\boldsymbol{z}_s(t)-\boldsymbol{u}_s(j)||^2/\sigma^2}} \tag{8}$$

Algorithm 1 Large-scale nonlinear Granger causality algorithm

1. Z-score the data, i.e. normalize the time-series to have zero mean and unit standard deviation. Focusing solely on system dynamics.
2. Using the order d, obtain phase space reconstructions of $\boldsymbol{Z}$. Here, $\boldsymbol{Z}$ is obtained using all the time-series in the system, $\boldsymbol{Z} \in \mathbb{R}^{Nd\times(T-d)}$.
3. From $\boldsymbol{Z}$, obtain c_f number of cluster centers with k-means clustering. The cluster centers are obtained using the mean of the samples in each cluster. Cluster centers $\boldsymbol{U}$ can be thought of as parameters of the hidden layer of a Generalized Radial Basis Function (GRBF) network having dimensions $Nd \times c_f$.
4. Set the width of the kernel as the mean distance between cluster centers.
5. Iterate through all N time-series from 1 to n where $n \in N$, selecting one time-series ($\mathbf{x}_s$) whose influence on other time-series is to be investigated.
   (a) Obtain phase space reconstructions $\boldsymbol{Z}_s \in \mathbb{R}^{(N-1)d\times(T-d)}$. States in this phase space do not contain information about $\mathbf{x}_s$.
   (b) Obtain $\boldsymbol{W}_s \in \mathbb{R}^{d\times(T-d)}$, the phase space reconstructions of $\mathbf{x}_s$, having embedding dimension d.
   (c) Obtain c_g number of cluster centers in the phase space $\boldsymbol{W}_s$ with k-means clustering. Set the width of the kernel as the mean distance between cluster centers. These parameters, $\boldsymbol{V}$, have dimensions $d \times c_g$.
   (d) Calculate activations for each of the c_g neurons using equation (7), given by $\mathbf{g}(\boldsymbol{W}_s)$, from each of the states in $\boldsymbol{W}_s$.
   (e) From $\boldsymbol{U}$, eliminate those dimensions corresponding to $\mathbf{x}_s$. For example, if $\mathbf{x}_s$ is the first time-series in the system, eliminate the first d dimensions of the cluster centers $\boldsymbol{U}$. In general, if it is the n-th time-series, eliminate d indices starting from and including $nd-(d-1)$. This results in cluster centers $\boldsymbol{U}_s$.
   (f) Calculate activations for each of the c_f neurons using equation (7), given by $\mathbf{f}(\boldsymbol{Z}_s)$, from each of the states in $\boldsymbol{Z}_s$.
   (g) **Predictions in the presence of $\mathbf{x}_s$**: Obtain $\hat{\mathbf{X}}$
   (h) **Predictions in the absence of $\mathbf{x}_s$**: Obtain $\tilde{\mathbf{X}}\backslash\mathbf{x}_s$
   (i) Calculate the influence of $\mathbf{x}_s$ on every time-series in $\mathbf{X}$ using the f-statistic.

3 Data

3.1 Complex system with three nodes:

The coupling parameters of this system are set such that they show fan-in and fan-out motifs.

$$x_j(t+1) = x_j(t)\Big(\gamma_{jj} - \sum_{i=1,2,3} \gamma_{ji} x_i(t)\Big), j = 1,2,3 \tag{9}$$

Here γ_{ji} are the coupling parameters. Again, we adopt parameter values from[9]. In the fan-out case, $\gamma_{11} = 4$, $\gamma_{22} = 3.1$, $\gamma_{33} = 2.12$, $\gamma_{21} = 0.21$ and $\gamma_{31} = -0.636$, the other parameters are zero (3-fan out). In the fan-in case, $\gamma_{11} = 4$, $\gamma_{22} = 3.6$, $\gamma_{33} = 2.12$, $\gamma_{31} = 0.636$ and $\gamma_{32} = -0.636$, the other parameters are zero (3-fan in). Uniformly distributed random numbers between [0, 1] are used as initial conditions and the first 50 time points are discarded.

3.2 5-node linear network

The linear implementation of interactions between the 5-node network time-series (whose network structure is the same as in example 3 of reference[10]) is provided here:

$$\begin{aligned}
x_1(t) &= x_1(t) + 0.95\sqrt{2}x_1(t-1) - 0.9025x_1(t-2) \\
x_2(t) &= x_2(t) + 0.5x_1(t-2) \\
x_3(t) &= x_3(t) - 0.4x_1(t-3) \\
x_4(t) &= x_4(t) - 0.5x_1(t-2) + 0.5\sqrt{2}x_4(t-1) + 0.25\sqrt{2}x_5(t-1) \\
x_5(t) &= x_5(t) - 0.5\sqrt{2}x_4(t-1) + 0.5\sqrt{2}x_5(t-1)
\end{aligned} \tag{10}$$

3.3 5-node nonlinear network:

Equations governing the non-linear 5-node network (5-nonlinear) are as follows:

$$
\begin{aligned}
x_1(t) &= x_1(t) + 0.95\sqrt{2}x_1(t-1) - 0.9025x_1(t-2)\\
x_2(t) &= x_2(t) + 0.5x_1^2(t-2)\\
x_3(t) &= x_3(t) - 0.4x_1(t-3)\\
x_4(t) &= x_4(t) - 0.5x_1^2(t-2) + 0.5\sqrt{2}x_4(t-1) + 0.25\sqrt{2}x_5(t-1)\\
x_5(t) &= x_5(t) - 0.5\sqrt{2}x_4(t-1) + 0.5\sqrt{2}x_5(t-1)
\end{aligned}
\tag{11}
$$

4 Results

4.1 5-node time-series results

The true connections of the 5 node network can be summarized as follows, $x_1 \to x_2$, $x_1 \to x_3$, $x_1 \to x_4$, $x_4 \to x_5$ and $x_5 \to x_4$, for the linear and non-linear case. LsNGC in the linear case clearly assigns high scores to the right connections, Figure 1. LsNGC correctly assigns a high score to $x_1 \to x_2$, $x_1 \to x_3$, $x_1 \to x_4$, $x_4 \to x_5$ and $x_5 \to x_4$ and assigns low scores to the rest of the connections which rightly correspond to non-existent connections. The high median AUC, specificity and sensitivity of 1, as shown in Figures 4 and 5 of the main paper, demonstrate that the correct network graph was recovered in most of the cases. LsNGC does not perform as well in the nonlinear case as compared to the linear one. Here, connections from $x_1 \to x_3$ and $x_4 \to x_5$ are detected well. However, the other connections were weaker. Quantitatively, this weak separation between the scores estimated by lsNGC for absence and presence of connections corresponds to the lower AUC, specificity and sensitivity values, as shown in Figures 4 and 5 of the main paper.

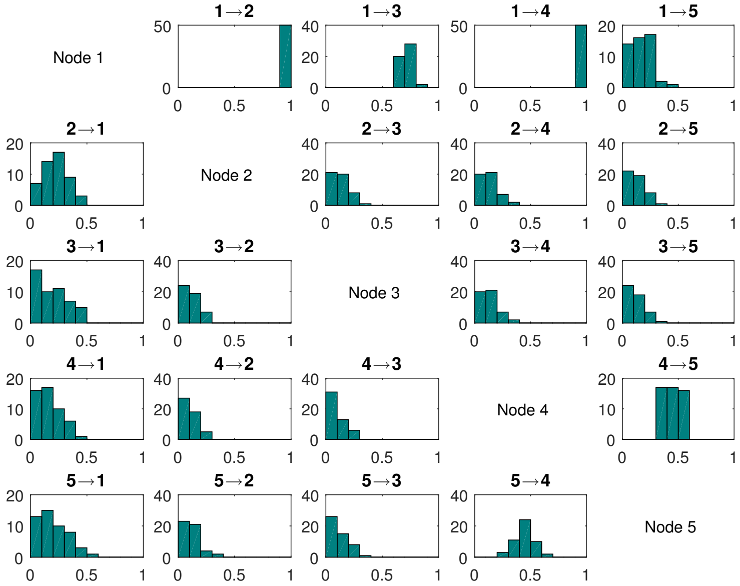

Figure 1. Histogram of scores (normalized between 0 to 1) obtained by lsNGC for the 5-node, linear (5-linear) network over 50 different sets of the simulation. Generated with MATLAB R2016a[11].

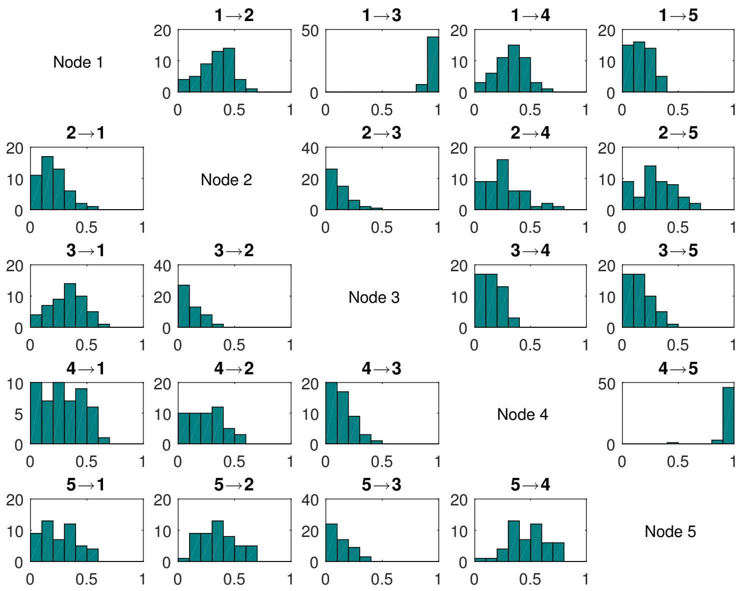

Figure 2. Histogram of scores (normalized between 0 to 1) obtained by lsNGC for the 5-node, nonlinear (5-nonlinear) network over 50 different sets of the simulation. Generated with MATLAB R2016a[11]

5 Comparative methods for evaluating lsNGC

In this section we briefly summarize the two comparison methods used to evaluate the performance of lsNGC.

5.1 Causality analysis with local models

An example for the use of local models (LM) to estimate casual relationships between time-series in a system is described in[12]. First, the attractor manifold of a time-series is constructed from time-delayed versions of itself and embedded into a state-space reconstruction (SSR). Such a space of embedded points transforms the observed time-series into a manifold[13,14] representing the evolution of states, i.e., its dynamics. The basic idea here is to build local models (using nearest neighbors) of the state space dynamics for every time-series, which cross-predicts the trajectory of an influencing time-series. Where, cross-prediction implies predicting say x_1 from the states of x_2. If x_2 cross-predicts x_1 well, then x_1 influences x_2.

5.2 Peter-Clark momentary conditional independence

The Peter and Clark momentary conditional independence (PCMCI) is a causal network inference method based on the graphical causal model framework[15] for large time-series featuring linear and nonlinear dependencies. The PC-based method, PCMCI, addresses the particular challenges of autocorrelated high-dimensional and nonlinear time series data based on a condition-selection step (PC), followed by the momentary conditional independence (MCI) test. The method is a non-parametric test designed for high-dimensional linear and nonlinear settings with time-delayed causal influences and strong autocorrelations. The PCMCI approach is also based on the conditional independence framework and adapts it to the highly interdependent time series case. The method consists of two stages: (i) PC condition selection to identify relevant conditions for parents of a node for all nodal time series variables and (ii) the momentary conditional independence (MCI) test to test whether there is a causal link between nodes[16,17]. In our simulations, we used partial correlation tests, and we used the toolbox available in PCMCI toolbox.

5.3 Multivariate transfer entropy

We use multivariate transfer entropy[18] with Kraskov nonlinear estimator[19] using the IDTxL toolbox[20]. The IDTxl employs a greedy or iterative approach that builds sets of parent sources for each target node in the network by maximizing a conditional mutual information criterion[21]. This iterative conditioning is designed to both remove redundancies and capture synergistic interactions in building each parent set. The conditioning thus automatically constructs a non-uniform, multivariate embedding of potential sources[21] and optimizes source-target delays[22]. Rigorous statistical controls (based on comparison to null

	lsNGC	LM	PCMCI	TE	KGC
2-Logistic	1, [1, 1]	1, [1, 1]	1, [1, 1]	1, [1, 1]	1, [1, 1]
3-Fan Out	1, [1, 1]	1, [1, 1]	1, [1, 1]	0.93, [0.93, 1]	1, [1, 1]
3-Fan In	1, [1, 1]	1, [1, 1]	1, [1, 1]	1, [1, 1]	1, [1, 1]
5-Linear	1, [1, 1]	0.87, [0.86, 0.88]	1, [1, 1]	1, [0.90, 1]	0.82, [0.76, 0.88]
5-Nonlinear	0.94, [0.90, 0.97]	0.62, [0.58, 0.67]	0.80, [0.72, 0.86]	0.95, [0.89, 0.99]	0.93, [0.91, 0.95]
34-Zachary1	0.79, [0.77, 0.81]	0.81, [0.79, 0.82]	0.74, [0.73, 0.76]	0.58, [0.57, 0.59]	0.53, [0.51, 0.56]
34-Zachary2	0.84, [0.82, 0.86]	0.88, [0.85, 0.89]	0.82, [0.79, 0.84]	0.72, [0.70, 0.74]	0.51, [0.48, 0.54]

Table 1. AUC values from Figure 4 of main manuscript. Each entry corresponds to Median AUC [25th percentile, 75th percentile] for all methods

distributions from time-series surrogates) are used to gate parent selection and provide automatic stopping conditions for the inference, requiring only a minimum of user-specified settings[20]. The code is from IDTxL toolbox. Based on the experimental analysis, we used the best parameters to maximize network discovery's success using multivariate transfer entropy on datasets.

5.4 Kernel Granger causality (KGC)

Kernel Granger causality (KGC) was proposed in 2008[23]. It calculates Granger causality in the feature space of kernel functions. In this work, we use radial basis function kernels for KGC.[10] We used the publicly available KGC toolbox to perform this analysis.

6 Estimating significance of connections from networks obtained

Measures of significance using lsNGC can be obtained from the f-statistic (equation (3)) and details regarding obtaining significance of connections with KGC can be found in[23]. Unlike lsNGC, KGC and PCMCI, LM and TE cannot be used directly to glean significance measures. Significance is calculated by establishing a null distribution, which was obtained by estimating LM (or TE) measures between non-interacting pairs of surrogate time-series. The surrogates used for LM were generated by using the Iterative Amplitude Adjusted Fourier Transform[24] algorithm generated with the Chaotic Systems Toolbox[25]. For TE, IDTxL provides the necessary software to perform such tests. Sensitivity and specificity were calculated on the connectivity matrix thresholded at $p < 0.05$, followed by multiple comparisons correction using False Discovery Rate, where the null hypothesis is generated by estimating connections between non-interacting surrogate time-series.

7 Application to fMRI data:

7.1 Resting-state fMRI data:

Functional MRI scans were obtained from human subjects at the Rochester Center for Brain Imaging (Rochester, NY, USA) using a 3T, Siemens Magneton TrioTim scanner. The study protocol included: (i) High-resolution structural imaging using T1-weighted magnetization-prepared rapid gradient echo sequence (MPRAGE, TE = 3.44 ms, TR= 2530 ms, isotropic voxel size 1 mm, flip angle = 7°). (ii) Resting-state fMRI scans using a gradient spin echo sequence (TE = 23 milliseconds, TR = 1650 milliseconds, 96 $\times$ 96 acquisition matrix, flip angle of 84°). The acquisition lasted 6 minutes and 54 seconds, and 250 temporal scan volumes were obtained. A total of 25 slices, each 5 mm thick, were acquired for each volume. During acquisition, the subject was asked to lie down still with eyes closed. The data were acquired as part of a NIH sponsored study (R01-DA-034977). Prior to computation of connectivity measures, the fMRI data used in this study was preprocessed using standard methodology. For each dataset, the first ten (of 250) volumes of functional magnetic resonance images were eliminated to analyze only those that reached steady-state imaging. Next, motion correction, brain extraction and correction for slice timing acquisition were performed. Additional nuisance regression was carried out to remove variations due to head motion and physiological processes. Each dataset was finally registered to the 2 mm MNI[26] standard space using a 12-parameter affine transformation[27]. All preprocessing steps were carried out using the C-PAC software[28] and its corresponding dependencies in FMRIB Software Library (FSL)[29]. Finally, the time-series were normalized to zero mean and unit standard deviation to focus on signal dynamics rather than amplitude[30]. Based on the commonly used Automated Anatomic Labeling (AAL) template[31], the registered MRI volumes were divided into 90 regions, excluding the brain stem and cerebellar regions, 45 in each hemisphere. A representative time-series for each region was computed by averaging the time-series of all voxels within it.

Subjects in this study were recruited as part of a NIH funded study (R01-DA-034977) at the University of Rochester Medical Center. In total, 15 healthy controls (mean age 42 $\pm$ 10 years) and 14 HIV positive subjects with symptoms of HIV associated neurocognitive disorder (HAND, mean age 45 $\pm$ 16 years) were recruited as part of this study. A standard battery of

neuropsychological (NP) tests was used to assess cognitive abilities of subjects, covering six cognitive domains: executive function, speed of information processing, attention, memory, learning, and motor function. These scores were converted to age and education adjusted z-scores. An overall z-score combining the scores from individual domains was generated and used to assess HAND[32]. All participants provided written informed consent prior to participation as per protocol approved by the institutional IRB.

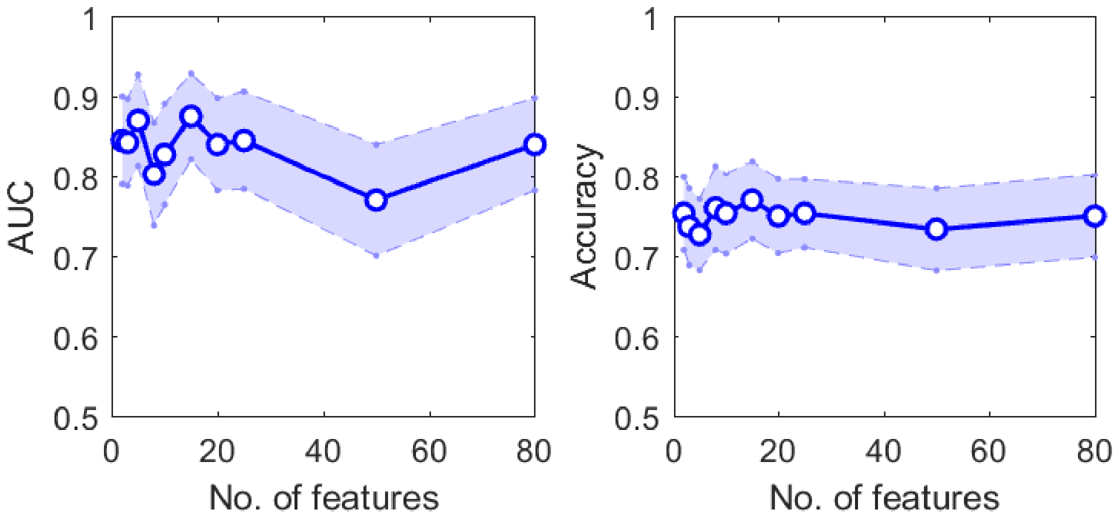

Figure 3. Plot of AUC and accuracy results for different number of retained features. Figure generated with MATLAB R2016a[11]. Shaded regions above and below each solid line, corresponding to the mean AUC/accuracy, represent the 95% confidence interval of the AUC/accuracy value. The general trend observed here is that lsNGC is able to discriminate between the two subject groups well.

7.2 Results

First, matrices of interaction using lsNGC, connectivity matrices, were obtained from the fMRI time-series of every subject in this study. LsNGC produced connectivity profiles conveying different information about interactions amongst regional time-series for every subject. These connectivity matrices were vectorized such that data from each subject was represented as an F dimensional vector of interactions, which can be viewed as features to train a classifier. Since, the number of features were large ($\sim$ 8000 for $N = 90$, where N is the number of regions defined by the AAL template) compared to the number of subjects in our study, before vectorizing the connectivity matrices, we symmetrized them such that $F = N(N-1)/2$, reducing the number of features to 4005 for each subject.

To further reduce the number of redundant and/or noisy features, we performed feature selection, using Kendall's τ correlation coefficient[33] . Feature selection aims at estimating interactions that best discriminate subjects presenting with HAND symptoms and controls and reduces the computational complexity of a classifier. Feature selection was performed independently for every set of training/test set split. Feature ranking estimated by Kendall's coefficient feature selection approach was used to select s features where $s \in \{2,3,5,8,10,15,20,25,50,80\}$.

The ranked features were classified with the AdaBoost[34] classifier. It is an ensemble classifier that uses an ensemble of weak classifiers, such as decision stump classifier, to produce a strong classifier. The WEKA[35] implementation of AdaBoost was imported into MATLAB 2016 and used in our analysis.

We ensured a strict split between training and testing data, with $90\%/10\%$ train/test separation within an iterative cross-validation scheme with 100 different data splits. The training data was used for both feature selection and training the classifier. Classification accuracy and the AUC were adopted to evaluate performance. An AUC of 1 indicates perfect classification while an AUC of 0.5 indicates random classification. The classifier for discriminating subjects with HAND and healthy controls achieved best mean AUC = 0.88 and accuracy = 0.77 with 15 features (Figure 3) which suggests that lsNGC was able to characterize the network structure well, hence the classifier was able to learn discriminate characteristics well.

References

1. Granger, C. W. Investigating causal relations by econometric models and cross-spectral methods. *Econom. J. Econom. Soc.* 424–438 (1969).
2. Ryali, S., Supekar, K., Chen, T. & Menon, V. Multivariate dynamical systems models for estimating causal interactions in fMRI. *NeuroImage* **54**, 807–823, DOI: 10.1016/j.neuroimage.2010.09.052 (2011).
3. Granger, C. W. J. Testing for causality. A personal viewpoint. *J. Econ. Dyn. Control.* **2**, 329–352, DOI: 10.1016/0165-1889(80)90069-X (1980).
4. Liao, W., Marinazzo, D., Pan, Z., Gong, Q. & Chen, H. Kernel Granger causality mapping effective connectivity on fmri data. *IEEE transactions on medical imaging* **28**, 1825–1835 (2009).
5. Marinazzo, D., Liao, W., Chen, H. & Stramaglia, S. Nonlinear connectivity by Granger causality. *Neuroimage* **58**, 330–338 (2011).
6. Li, X., Marrelec, G., Hess, R. F. & Benali, H. A nonlinear identification method to study effective connectivity in functional mri. *Med. image analysis* **14**, 30–38 (2010).
7. Moody, J. & Darken, C. J. Fast learning in networks of locally-tuned processing units. *Neural computation* **1**, 281–294 (1989).
8. Ancona, N., Marinazzo, D. & Stramaglia, S. Radial basis function approach to nonlinear Granger causality of time series. *Phys. Rev. E* **70**, 056221 (2004).
9. Ma, H., Aihara, K. & Chen, L. Detecting causality from nonlinear dynamics with short-term time series. *Sci. reports* **4** (2014).
10. Baccalá, L. A. & Sameshima, K. Partial directed coherence: a new concept in neural structure determination. *Biol. cybernetics* **84**, 463–474 (2001).
11. The Mathworks, Inc., Natick, Massachusetts. *MATLAB version 9.0.0.341360 (R2016a)* (2016).
12. Sugihara, G. *et al.* Detecting causality in complex ecosystems. *Science* **338**, 496–500 (2012).
13. Takens, F. Detecting strange attractors in turbulence. In *Dynamical systems and turbulence, Warwick 1980*, 366–381 (Springer, 1981).
14. Deyle, E. R. & Sugihara, G. Generalized theorems for nonlinear state space reconstruction. *PLoS One* **6**, e18295 (2011).
15. Spirtes, P., Glymour, C. N., Scheines, R. & Heckerman, D. *Causation, prediction, and search* (MIT press, 2000).
16. Runge, J., Nowack, P., Kretschmer, M., Flaxman, S. & Sejdinovic, D. Detecting and quantifying causal associations in large nonlinear time series datasets. *Sci. Adv.* **5**, eaau4996 (2019).
17. Runge, J. *et al.* Inferring causation from time series in earth system sciences. *Nat. communications* **10**, 1–13 (2019).
18. Schreiber, T. Measuring information transfer. *Phys. review letters* **85**, 461 (2000).
19. Kraskov, A., Stögbauer, H. & Grassberger, P. Estimating mutual information. *Phys. review E* **69**, 066138 (2004).
20. Wollstadt, P. *et al.* Idtxl: The information dynamics toolkit xl: a python package for the efficient analysis of multivariate information dynamics in networks. *arXiv preprint arXiv:1807.10459* (2018).
21. Faes, L., Nollo, G. & Porta, A. Information-based detection of nonlinear granger causality in multivariate processes via a nonuniform embedding technique. *Phys. Rev. E* **83**, 051112 (2011).
22. Wibral, M. *et al.* Measuring information-transfer delays. *PloS one* **8**, e55809 (2013).
23. Marinazzo, D., Pellicoro, M. & Stramaglia, S. Kernel-Granger causality and the analysis of dynamical networks. *Phys. review E* **77**, 056215 (2008).
24. Schreiber, T. & Schmitz, A. Improved surrogate data for nonlinearity tests. *Phys. Rev. Lett.* **77**, 635 (1996).
25. Leontitsis, A. Chaotic systems toolbox, https://www.mathworks.com/matlabcentral/fileexchange/1597-chaotic-systems-toolbox (2020).
26. Mazziotta, J. *et al.* A probabilistic atlas and reference system for the human brain: International Consortium for Brain Mapping (ICBM). *Philos. Transactions Royal Soc. London. Ser. B: Biol. Sci.* **356**, 1293–1322 (2001).
27. Jenkinson, M. & Smith, S. A global optimisation method for robust affine registration of brain images. *Med. image analysis* **5**, 143–156 (2001).

28. Sikka, S. *et al.* Towards automated analysis of connectomes: The configurable pipeline for the analysis of connectomes (c-pac). In *5th INCF Congress of Neuroinformatics, Munich, Germany*, vol. 10 (2014).

29. Smith, S. M. *et al.* Advances in functional and structural MR image analysis and implementation as FSL. *NeuroImage* **23 Suppl 1**, S208–19, DOI: 10.1016/j.neuroimage.2004.07.051 (2004).

30. Wismüller, A. *et al.* Cluster analysis of biomedical image time-series. *Int. J. Comput. Vis.* **46**, 103–128, DOI: 10.1023/A:1013550313321 (2002).

31. Tzourio-Mazoyer, N. *et al.* Automated anatomical labeling of activations in SPM using a macroscopic anatomical parcellation of the MNI MRI single-subject brain. *Neuroimage* **15**, 273–289 (2002).

32. Antinori, A. *et al.* Updated research nosology for HIV-associated neurocognitive disorders. *Neurology* **69**, 1789–1799 (2007).

33. Kendall, M. G. Rank correlation methods (1955).

34. Freund, Y. & Schapire, R. E. A decision-theoretic generalization of on-line learning and an application to boosting. *J. computer system sciences* **55**, 119–139 (1997).

35. Hall, M. *et al.* The WEKA data mining software: an update. *ACM SIGKDD explorations newsletter* **11**, 10–18 (2009).
